# Pathology and Histology

**Published:** 1876-06

**Authors:** 


### V. Pathology and Histology.

1. *Amyloid Degeneration of the Muscular Structure of the Heart.* Prof. Heschl. (*Wiener Med. Wochenschr.*, 1876. No. 2.)

Some time ago Prof. Heschl, of Vienna, recommended Leonard's violet ink as a very delicate test for the amyloid degeneration of tissues (see Chicago Medical Journal and Examiner, December, 1875) And now he can give another proof of the superior delicacy of this new test. Though often suspecting an amyloid state of the heart's muscle he had never been able by the ordinary tests to verify his suspicion; while now with the aid of the violet ink, Dr. Breus has detected the amyloid degeneration of the heart in a case of caries of the spine with amyloid degeneration of the liver, spleen and kidneys. Smaller and larger arteries, sometimes the capillaries too, and the connective tissue surrounding them, gave the characteristic reaction. As to the muscular fibres the primitive bundles themselves are unaltered, but they are enveloped by a homogeneous substance giving the characteristic reaction of amyloid matter. As the bundles of the heart muscle have no sarcolemma, the above described amyloid substance surrounding the muscular fibrillæ must be a new formation and most likely of an exudative origin, because of the want of texture.

